# Severe immune checkpoint inhibitor-induced 3M syndrome: a case report

**DOI:** 10.3389/fimmu.2026.1840526

**Published:** 2026-05-29

**Authors:** Hairong Yao, Zitong Hao, Xin Zhang, Shikai Liu

**Affiliations:** Department of Gynecologic Oncology, Cangzhou Central Hospital, Cangzhou, Hebei, China

**Keywords:** 3M syndrome, cervical cancer, early monitoring, immune checkpoint inhibitor-associated myocarditis, immune checkpoint inhibitors

## Abstract

**Background:**

Immune checkpoint inhibitors (ICIs) have significantly improved survival rates in various advanced cancers, including cervical cancer. However, they can induce a range of immune-related adverse events (irAEs), with immune myocarditis being particularly dangerous, especially when combined with myositis and myasthenia gravis, forming the fatal 3M syndrome. Early symptoms are subtle, and delayed diagnosis and treatment often lead to severe consequences.

**Case presentation:**

A 65-year-old female with stage FIGO stage IIIA cervical squamous cell carcinoma received one dose of anti-PD-1 monoclonal antibody (Enlarzumab) combined with chemoradiotherapy. Following initial disease stabilization, the patient developed early neuromuscular symptoms, including ptosis and facial weakness, on Day 21. By Day 25, she experienced sudden chest tightness and acute heart failure with new-onset atrial fibrillation and a decline in left ventricular ejection fraction from 57% to 40%. The concurrent elevation of cardiac biomarkers and creatine kinase, alongside neuromuscular involvement, confirmed the clinical diagnosis of ICI-induced 3M syndrome. Despite treatment with high-dose methylprednisolone, intravenous immunoglobulin (IVIG), and mycophenolate mofetil, the patient ultimately succumbed to progressive respiratory failure following the family’s decision to decline further invasive respiratory support.

**Conclusion:**

This case highlights the insidious onset and rapid progression of 3M syndrome, where neuromuscular symptoms often serve as overlooked precursors to myocarditis. Establishing a systematic approach involving proactive monitoring and immediate immunosuppressive treatment is essential to ensure the safety of immunotherapy.

## Introduction

Immune checkpoint inhibitors (ICIs), particularly those targeting programmed cell death protein 1 (PD-1) and its ligand PD-L1, have transformed the therapeutic landscape of advanced cervical cancer, offering meaningful survival benefits for patients with recurrent or metastatic disease (1). By restoring T-cell–mediated antitumor immunity, these agents improve clinical outcomes but may also disrupt immune tolerance. This can lead to immune-related adverse events (irAEs) affecting multiple organs; among these, the overlap of neuromuscular and cardiovascular toxicities is particularly life-threatening (2).

Although cardiovascular irAEs are relatively uncommon, with an estimated incidence of 1–2%, they are associated with disproportionately high mortality (3). Among these, immune checkpoint inhibitor–associated myocarditis represents the most severe manifestation. Notably, myocarditis frequently coexists with myositis and myasthenia gravis (MG), forming the so-called “3M syndrome.” This overlapping syndrome is characterized by early onset, typically within weeks after initiation of ICIs, rapid clinical deterioration, diagnostic complexity, and markedly elevated mortality rates, reported to be as high as 40–60% (4–6).

With the expanding use of ICIs in gynecologic malignancies, particularly cervical cancer, recognition of rare but life-threatening toxicities such as 3M syndrome has become increasingly important. However, early manifestations are often nonspecific, including fatigue, mild muscle weakness, ptosis, or dizziness, and may be easily misattributed to disease progression, treatment-related toxicity, or benign conditions. Such diagnostic challenges frequently result in delayed intervention and poor outcomes. Although current international guidelines have proposed management strategies for ICI-related myocarditis (2, 7), standardized approaches for early detection and risk stratification, especially in the context of overlapping syndromes, remain insufficiently defined.

Herein, we report a case of 3M syndrome following anti-PD-1 therapy in a patient with cervical cancer. By analyzing the missed warning signs and delays in intervention, this report aims to provide practical guidance for clinicians on structured monitoring and emergency management strategies to optimize treatment for this life-threatening complication.

## Case presentation

### Patient information and baseline assessment

A 65-year-old female was admitted (Day -12) with a two-year history of postmenopausal bleeding. Gynecological examination revealed loss of normal cervical architecture with a volcano-shaped lesion involving the vaginal wall. Cervical biopsy confirmed squamous cell carcinoma. Pelvic magnetic resonance imaging (MRI) demonstrated invasion of the right vaginal fornix and wall, diagnosed as Federation International of Gynecology and Obstetrics (FIGO) stage IIIA cervical squamous carcinoma.

Her medical history included facial nerve palsy over 30 years earlier, with complete recovery. She had no history of hypertension, coronary artery disease, or diabetes mellitus. Baseline echocardiogram showed a left ventricular ejection fraction (LVEF) of 57% with mild mitral and tricuspid and mitral regurgitation. Electrocardiogram (ECG) and cardiac biomarkers were within normal limits.

### Initial treatment

The patient received standard concurrent chemoradiotherapy. Radical pelvic external beam radiotherapy was initiated on Day -12. Concurrent chemotherapy with vinorelbine (60 mg) was administered on Days 0, 7, and 14. In addition, immunotherapy with Enlarzumab (360 mg) was initiated on Day 0, following the first cycle of chemotherapy.

### Early immune-related adverse events

Approximately 20 days after initiation of immunotherapy, the patient developed endocrine abnormalities. On Day 20, as part of the routine proactive surveillance prior to the next scheduled cycle of immunotherapy, laboratory testing revealed thyroid dysfunction. The results showed suppressed thyroid-stimulating hormone (TSH, 0.02 mIU/L, reference range: [0.27-4.2] mIU/L) and elevated free triiodothyronine (FT3, 7.81 pmol/L, reference range: [2.0-4.4] pg/mL) and free thyroxine (FT4, 5.13 pmol/L, reference range: [0.93-1.7] ng/dl). Thyrotropin receptor antibody (TRAb) was positive (1.76 IU/L, reference range: <115IU/ml). The patient remained asymptomatic, and immune-related hyperthyroidism was suspected. Close observation was adopted.

### Neuromuscular manifestations and hepatic injury

On Day 21, the patient developed dizziness, blurred vision, and a fall resulting in facial abrasion. Brain computed tomography showed no acute abnormalities. Neurological symptoms progressed, including ptosis and deviation of the mouth. Given the concurrent chemotherapy regimen, neurotoxicity from vinorelbine was briefly considered but deemed unlikely due to the rapid onset of unilateral ptosis and facial involvement. Acute ischemic stroke was a primary concern, but the brain computed tomography showed no acute hemorrhage or infarction. Central nervous system infection was also considered less likely in the absence of fever or meningeal signs. Unfortunately, during this early phase, these symptoms were managed as an isolated cerebrovascular or vestibular event by the neurology consultation, and only symptomatic treatment was provided without linking them to the recently initiated ICI.

On Day 22, magnetic resonance angiography revealed arteriosclerosis and severe stenosis of the right posterior cerebral artery. Meanwhile, liver function tests showed significant elevation of transaminases (ALT 304.8 U/L, AST 412.9 U/L) and bilirubin. To systematically evaluate concurrent hepatic enzyme elevations and rule out pre-existing autoimmune liver diseases, a comprehensive autoimmune serology panel was ordered. This panel revealed positive results for antinuclear antibody (ANA) and gp210 antibody. Imaging suggested mild fatty liver. Immune-related hepatitis was suspected, and hepatoprotective therapy was initiated.

### Acute cardiac involvement and diagnosis

On Day 25, the patient developed chest tightness and palpitations. ECG revealed atrial fibrillation with rapid ventricular response (~140 bpm), left anterior fascicular block, and incomplete right bundle branch block. Cardiac biomarkers were markedly elevated (troponin I 3.94 ng/mL, creatine kinase 5036 U/L). Echocardiogram showed a decline in LVEF to 54%.

At this critical juncture, an emergency multidisciplinary team (MDT) including cardiology and critical care was activated. Acute coronary syndrome was rapidly ruled out given the absence of prior coronary artery disease and the fulminant nature of the cardiac marker elevation. The definitive diagnosis of ICI-induced 3M syndrome was established based on the classic clinical and biochemical triad: 1) acute myocarditis (markedly elevated troponin I, new-onset arrhythmias, and reduced LVEF), 2) myositis (creatine kinase soaring to 5036 U/L), and 3) myasthenia-like features or severe bulbar myositis (progressive ptosis and bulbar weakness leading to respiratory failure). Due to the patient’s rapid hemodynamic collapse, transporting the patient for electromyography (EMG) was clinically unsafe. Furthermore, specific acetylcholine receptor (AChR) antibody serology, which requires outsourcing to an external laboratory, was unfortunately not obtained due to the extreme urgency of the resuscitation and the lack of a sufficient time window before the patient’s rapid demise. Therefore, while pure neuromuscular junction involvement cannot be definitively distinguished from severe ICI-induced myopathy without these tests, the clinical presentation strongly indicated an ICI-induced overlap syndrome.

**Figure 1:**
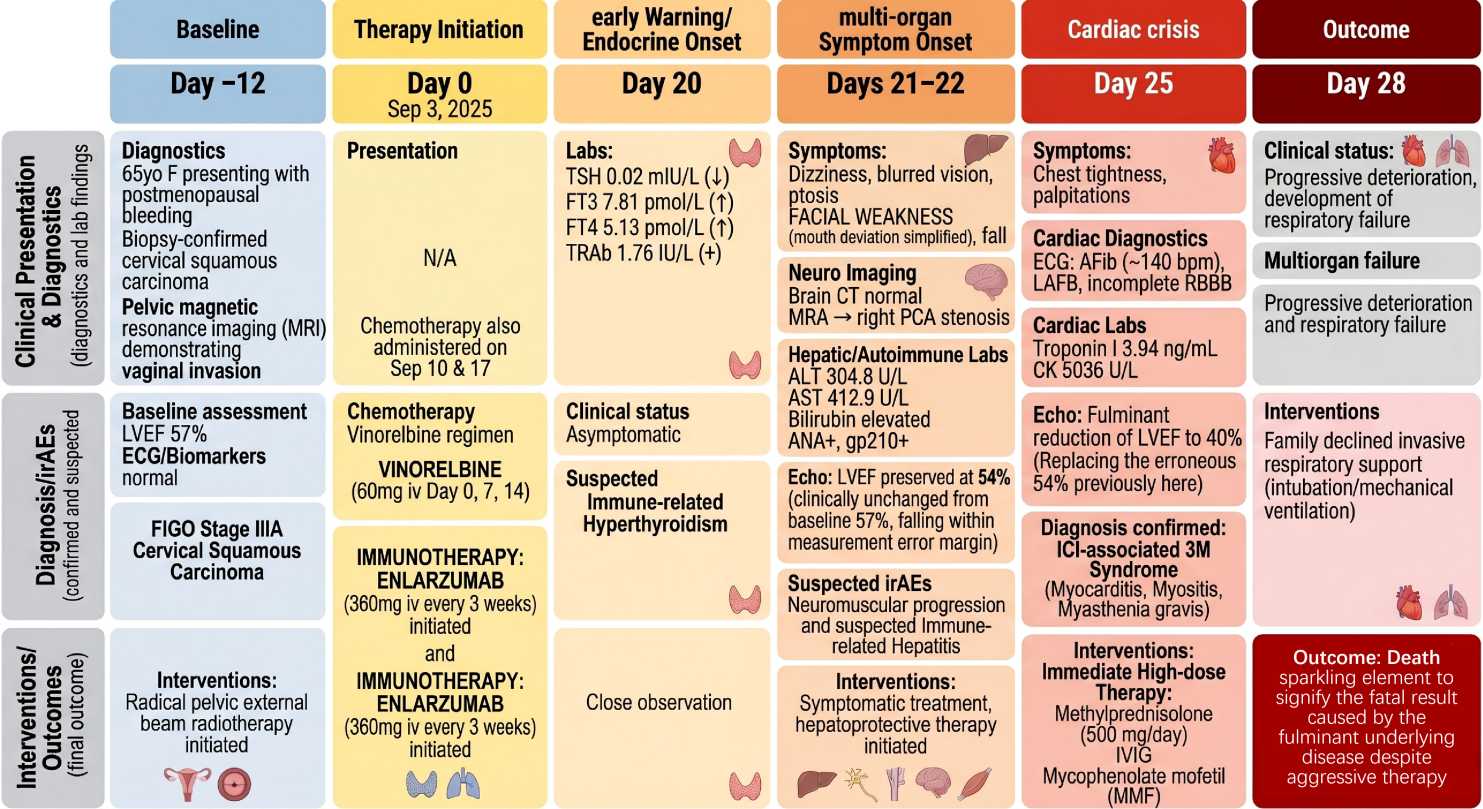
Timeline chart illustrating the progression of a 65-year-old female patient with cervical squamous carcinoma, detailing key clinical findings, diagnostics, therapy initiation, adverse events, and multi-organ failure leading to fatal outcome over a 28-day period, including interventions and immune therapy.

The detailed chronological sequence of the patient’s clinical course, including disease presentation, symptom onset, diagnostic workup, interventions, and ultimate outcome, is summarized in the structured clinical timeline (**Figure 1**).

### Treatment and clinical outcome

High-dose methylprednisolone (500 mg/day) was initiated immediately, along with intravenous immunoglobulin (IVIG) and mycophenolate mofetil (MMF). Although cardiac biomarkers showed initial improvement, the patient’s clinical condition progressively deteriorated with the development of respiratory failure. On Day 28, following extensive discussions regarding the poor prognosis, the family declined invasive respiratory support (intubation and mechanical ventilation), and the patient passed away.

## Discussion

Immune checkpoint inhibitor–associated 3M syndrome represents a rare but highly fatal complication characterized by the overlap of myocarditis, myositis, and myasthenia gravis. This case report describes a fatal ICI-induced 3M syndrome in a patient with cervical cancer and highlights the catastrophic consequences of missed early warning signs and delayed intervention. The clinical course followed a sequential pattern. It began with hyperthyroidism, progressing to neuromuscular manifestations and liver injury, and ultimately advancing to an acute cardiac crisis. This progression reflects the typical trajectory of this overlapping immune-related adverse event syndrome.

This case underscores the key clinical features of 3M syndrome. While isolated irAEs are common, the simultaneous and profound breakdown of immune tolerance affecting the endocrine, hepatic, neuromuscular, and cardiac systems in a single patient is exceptionally rare, reflecting a catastrophic systemic ‘immune storm’. This rapid progression and extensive multisystem involvement culminated in a fulminant and severe outcome despite the initiation of immunosuppressive therapy at an advanced stage.

While ICI-induced 3M syndrome is increasingly recognized in melanoma and thoracic malignancies, its occurrence in gynecologic oncology remains exceptionally rare. To our knowledge, only a few isolated cases of 3M syndrome have been reported in gynecologic cancers, such as a recently documented case in endometrial cancer (8). This highlights the significant novelty of our report, which presents one of the first detailed accounts of fulminant 3M syndrome triggered by an anti-PD-1 inhibitor combined with concurrent chemoradiotherapy in locally advanced cervical cancer. Comparing our case with these emerging reports in gynecologic oncology reveals striking similarities in diagnostic challenges: early neuromuscular symptoms (such as ptosis) are frequently misattributed to general fatigue, chemoradiotherapy toxicity, or unrelated neurological events. This emerging clinical pattern underscores the urgent need for heightened awareness and centralized multidisciplinary team (MDT) management specifically tailored for patients undergoing ICI therapies in gynecologic oncology.

A key lesson from this case is that the fatal outcome in this case was not due to a lack of therapeutic options, but rather to delayed recognition of an evolving systemic immune-mediated process. The patient developed a sequence of immune-related adverse events, including hyperthyroidism, neuromuscular symptoms (ptosis and facial weakness), and hepatic injury. However, these manifestations were managed as isolated conditions rather than being integrated into a unified diagnosis of systemic immune toxicity. Notably, neuromuscular symptoms, particularly ptosis as a hallmark feature of myasthenia gravis, have been reported as early warning signs preceding ICI-associated myocarditis and should prompt immediate evaluation for overlapping syndromes (9, 10).

Studies demonstrate that neuromuscular symptoms precede cardiac manifestations in approximately 70% of 3M syndrome cases, with a median interval of 3–7 days between symptom onset and cardiac decompensation (11, 12). This 4-day interval (Days 21–25) represents a critical missed opportunity. What could have been done earlier was the immediate suspension of the ICI and the empirical initiation of high-dose corticosteroids at the very first sign of new-onset ptosis, rather than waiting for overt cardiac decompensation. The failure to connect these sequentially emerging, seemingly isolated organ toxicities into a unified diagnosis of systemic immune hyperactivation ultimately allowed the irreversible progression of myocardial injury.

The timing of intervention is critical in determining prognosis. In this case, there was a delay of several days between the onset of neuromuscular symptoms and the initiation of high-dose corticosteroid therapy. Emerging evidence strongly supports the early administration of high-dose corticosteroids, ideally within 24 hours of suspected myocarditis. Such prompt intervention is associated with improved outcomes, whereas delays significantly increase mortality risk (13). This underscores the importance of maintaining a high index of suspicion and initiating empirical immunosuppressive therapy when 3M syndrome is suspected, even before definitive confirmation.

Reflecting on our patient’s rapid deterioration, there is an urgent need to shift from passive management of irAEs to proactive surveillance. If a structured early monitoring strategy, such as serial measurement of high-sensitivity troponin and regular ECG screening during her high-risk window (the first 4–6 weeks), had been employed, her acute cardiac crisis on Day 25 might have been anticipated. Furthermore, comprehensive baseline cardiovascular and neuromuscular evaluations are necessary to identify high-risk patients before ICI initiation (14, 15). During the first 4 to 6 weeks, which is recognized as the high-risk window, close monitoring is crucial (16). Serial measurement of cardiac biomarkers, particularly high-sensitivity troponin, along with regular electrocardiogram screening, should be incorporated into routine clinical practice. Importantly, the emergence of even mild or nonspecific symptoms, such as fatigue, ptosis, or palpitations, should trigger immediate and thorough evaluation to exclude early myocarditis or overlapping syndromes (17, 18).

Furthermore, our case highlights the critical need for an immediate, standardized therapeutic response. When our patient presented with dizziness and ptosis on Day 21, prompt implementation of high-dose corticosteroids combined with intravenous immunoglobulin, rather than waiting until overt heart failure developed on Day 25, could have potentially halted the immune storm (19). For patients with severe or refractory disease, early escalation to additional immunosuppressive agents, such as mycophenolate mofetil or cyclophosphamide, may be necessary (20, 21). Importantly, permanent discontinuation of immune checkpoint inhibitors is recommended in cases of severe cardiac toxicity.

Crucially, this case reflects the real-world challenges of fragmented medical care. During the early symptom onset (Days 20-22), consultations from endocrinology, neurology, and gastroenterology occurred in isolation. It was not until the acute cardiac crisis on Day 25 that a comprehensive MDT involving cardiology and critical care was fully mobilized. This emphasizes that for patients on ICIs, new-onset multisystem symptoms must immediately trigger a centralized, proactive MDT response rather than sequential single-specialty consults to facilitate early recognition and coordinated management. To synthesize the practical clinical lessons from this case, we propose three actionable strategies. First, clinicians must conduct comprehensive baseline cardiovascular and neuromuscular assessments before ICI initiation to identify subclinical risks. Second, during the high-risk window (the first 4–6 weeks), management must shift from passive observation to proactive surveillance, incorporating routine serial high-sensitivity troponin and ECG monitoring. Third, the onset of seemingly ‘isolated’ endocrine abnormalities or neuromuscular symptoms (such as ptosis) should immediately trigger a centralized multidisciplinary team (MDT) response and ICI suspension, rather than waiting for fulminant cardiac symptoms to emerge.

In conclusion, ICI-associated 3M syndrome is a rapidly progressive condition requiring heightened clinical awareness. Implementing a proactive, system-based approach to the management of immune-related adverse events is imperative to improving patient survival in the era of cancer immunotherapy.

## Data Availability

The raw data supporting the conclusions of this article will be made available by the authors, without undue reservation.
